# Pachydermodactyly Treated with Tranilast in a Young Girl

**DOI:** 10.1155/2014/132854

**Published:** 2014-08-06

**Authors:** Chikahisa Higuchi, Tetsuya Tomita, Hideki Yoshikawa

**Affiliations:** Department of Orthopaedic Surgery, Osaka University Graduate School of Medicine, 2-2 Yamadaoka, Suita, Osaka 565-0871, Japan

## Abstract

*Introduction.* Pachydermodactyly is a rare disease with asymptomatic swelling of proximal interphalangeal joints. This disorder should be considered in the differential diagnosis of juvenile idiopathic arthritis or rheumatoid arthritis. However, pachydermodactyly is not well recognized by many orthopaedic surgeons and rheumatologists. *Case Presentation.* We report herein a case of a 13-year-old girl with pachydermodactyly. She presented to our clinic with symmetrical swelling of digits II through V without functional loss for the last 4 years. X-ray examination demonstrated no bone or joint destruction and magnetic resonance images showed only thickened skin tissues. No inflammatory signs were seen with laboratory blood tests. We reached a diagnosis of pachydermodactyly by exclusion. We had administered tranilast to her for 6 months and her symptom slightly improved. *Conclusion.* It is important to recognize pachydermodactyly and be able to differentiate it from other causes of PIP joint swelling such as rheumatoid arthritis, although pachydermodactyly is rare and benign. Physicians including orthopaedists and rheumatologists should make a prompt diagnosis to avoid unnecessary investigations and prevent the patient from receiving inappropriate treatment with steroids or cytotoxic agents. On the other hand, tranilast might be an effective drug to pachydermodactyly.

## 1. Introduction

Pachydermodactyly is a rare disease with noninflammatory swelling around the proximal interphalangeal (PIP) joints of digits II–IV. The thumb or digit V is sometimes affected. X-ray investigation demonstrates no bony or articular changes. Several papers reported that the affected lesion was histologically composed of hyperkeratosis and that increased collagen was present in dermal tissues [1–6].

This disease is clinically characterized by asymptomatic PIP joint swelling. Therefore, it is necessary for physicians, especially orthopaedic specialists and rheumatologists, to distinguish this disease from juvenile idiopathic arthritis for which appropriate treatment can be provided. However, most physicians rarely encounter pachydermodactyly and have difficulty recognizing the disease.

Tranilast is clinically used as an antiallergic drug. It is also used for the treatment of keloid formation because it inhibits collagen synthesis in skin [7]. However, there are no reports of treatment with tranilast for pachydermodactyly.

We herein reported a case of a 13-year-old girl with pachydermodactyly. Additionally, we presented our experience of administration of tranilast to pachydermodactyly.

## 2. Case Presentation

**Figure 1:**
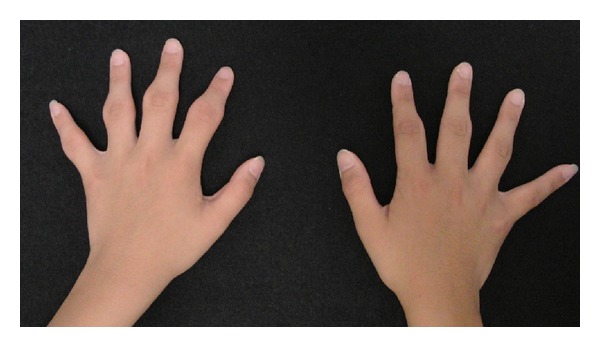
Photographic image of bilateral hands. Symmetrical swelling around PIP joints of bilateral digits II–V.

**Figure 2:**
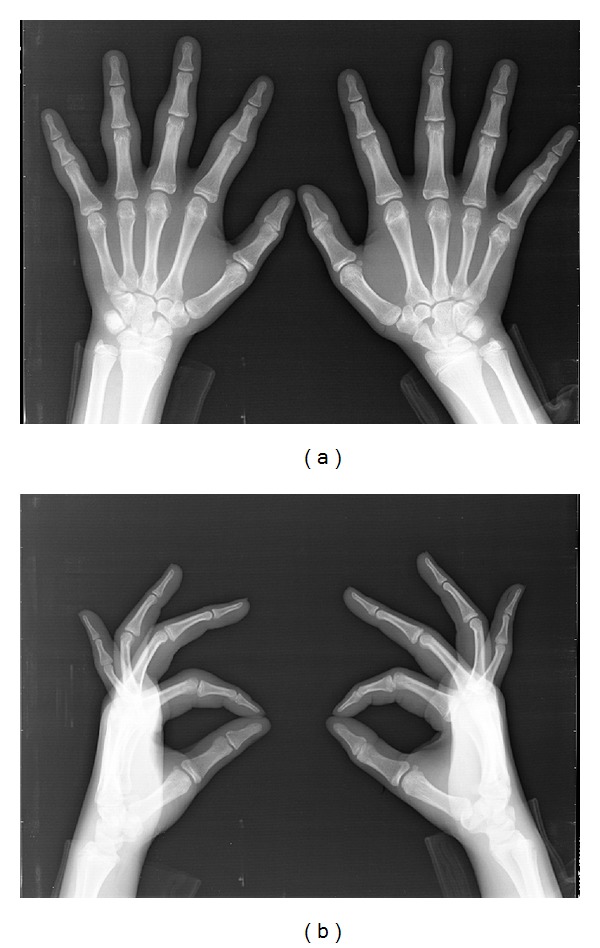
Plain radiograph of bilateral hands at the first examination. No bony erosive and joint destruction around the PIP joints on anteroposterior (a) and lateral (b) images.

**Figure 3:**
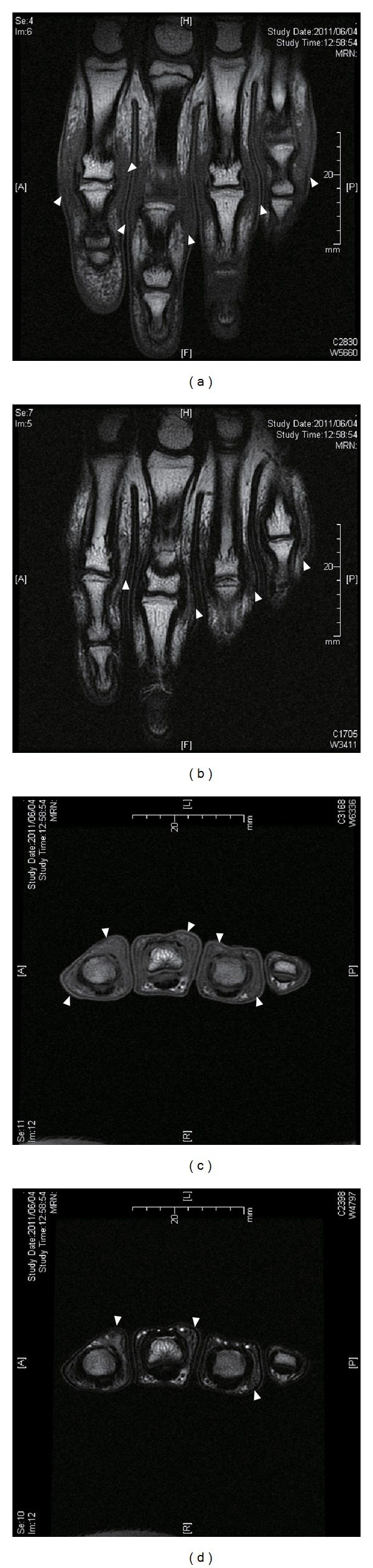
Coronal (a, b) and axial (c, d) views of magnetic resonance imaging of the left hand. Homogeneous low-intensity subcutaneous lesion around the PIP joints (arrow heads) on T1-weighted image (a, c) and T2-weighted image (b, d).

**Figure 4:**
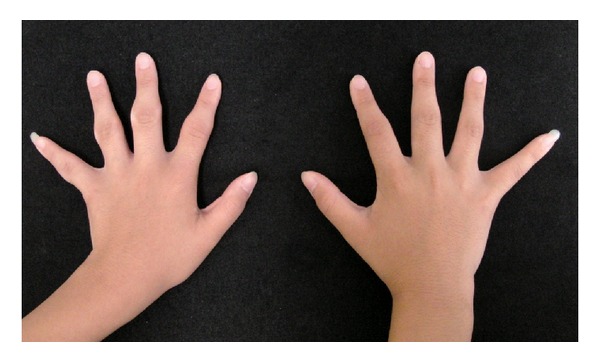
Photographic image of bilateral hands after treatment with tranilast. Improvement of symmetrical swelling around PIP joints after treatment with tranilast.

A 13-year-old girl presented with swelling surrounding the PIP joints of both hands since she was 9 years old. She had slight pain surrounding the PIP joints on her first visit to our clinic. She and her mother both reported no history of trauma or microtrauma. Our first physical examination revealed only symmetrical thickening around the PIP joints of bilateral digits II–V. She experienced slight pain surrounding the affected lesions. However, there was no restriction of finger range of motion and there was no disturbance of activities of daily life (Figure 1). Laboratory investigation results, including white and red blood cell counts, liver function, rheumatoid factor, C-reactive protein, matrix-metalloprotease 3, antinuclear antibodies, and anti-cyclic citrullinated peptide (CCP) antibodies, were within normal limits. Radiography of both hands showed no joint space narrowing and no evidence of bony erosive change around the PIP joints (Figure 2). Magnetic resonance imaging (MRI) revealed only soft tissue thickness surrounding the PIP joints without bony change (Figure 3). Full analysis of the test results yielded a diagnosis of pachydermodactyly. The patient and her mother were educated on the nature of her disease, explaining that it was not rheumatic in nature and did not require aggressive treatment. We initiated nonsteroidal anti-inflammatory drug (NSAID) therapy for her irritative pain around PIP joints. After good pain control, she took 300 mg of tranilast daily for 6 months. Her affected digits slightly softened and became less swelled at the last visit to our clinic (Figure 4).

## 3. Discussion

Pachydermodactyly was first reported as fibromatosis and hyperplastic dermis of digits II–IV by Bazex in 1973 [8]. Verbov named this entity as pachydermodactyly from the Greek* pachy* (thick),* dermo* (skin), and* dactyly* (finger) in 1975 [9]. There have been less than 100 cumulative reports of pachydermodactyly cases worldwide [5]. One review showed that the ratio of males to females was 5 : 1 [10]. However, some authors have argued that the female prevalence may be underestimated. A recent review of pachydermodactyly literature suggests that it has a male-dominant tendency with a male/female ratio of 3/2 [11].

The etiology of pachydermodactyly is unclear. Some papers have inferred that there is a relationship between repetitive mechanical skin stimulation and the pathogenesis of pachydermodactyly [9]. In our case, no history of repetitive trauma was found and the etiology was not understood.

Several groups indicated that the histopathological features of pachydermodactyly include dermal collagen accumulation accompanied by hyperkeratosis and fibroblast proliferation with no inflammatory infiltration [8, 9]. Another group showed that the affected skin lesion consists of collagen types III and V, which differs from the collagen profile of normal skin [2]. Some authors reported a decreased collagen fiber diameter [12] and several reports indicated that a histological examination is not necessary to make the diagnosis of this disease [13]. Skin biopsy procedures are invasive and we did not undergo this investigation.

Pachydermodactyly is clinically characterized by asymptomatic bulbous swelling of the lateral aspects of the PIP joints of digits II–IV. As a rule, the appearance is bilaterally symmetric. There are no underlying bony abnormalities, synovitis, or joint movement limitations associated with pachydermodactyly. X-ray images of the hands are normal. Magnetic resonance imaging of affected fingers shows typical fusiform soft tissue swelling around the PIP joints [14]. Our patient presented with symptoms that were consistent with most of the criteria of this disease. However, she had finger pain and both digits V were affected, which is not typical. As her pain was controllable by NSAIDs, we regarded her pathology as pachydermodactyly.

The differential diagnosis of pachydermodactyly includes Thiemann disease, pachydermoperiostitis, knuckle pads, infectious diseases, arthritis, and tumors around the fingers. In particular, pachydermodactyly must be considered in the differential diagnosis of juvenile idiopathic arthritis and rheumatoid arthritis because symmetrical PIP joint swelling is one of the most typical symptoms in the onset of these rheumatic diseases. However, it is not difficult to make a definite diagnosis of pachydermodactyly. The diagnosis of pachydermodactyly can be made with routine laboratory and radiological assessment. Laboratory investigations reveal negative erythrocyte sedimentation rate and C-reactive protein (CRP). In addition, antinuclear antibodies and serum rheumatoid factor are negative. Laboratory values for our case also revealed negative anti-CCP antibodies, which is highly sensitive and specific for rheumatic diseases. Moreover, normal matrix metalloproteinase 3 was observed in our case, indicating a low possibility of rheumatoid arthritis because of a lack of evidence for joint cartilage injury. It is important for physicians to recognize this disease.

There are no papers of effective treatment for swelling of affected fingers. Almost all published papers suggest that it is not necessary to administer medication in the treatment of pachydermodactyly. Our patient was treated with NSAIDs for her irritating skin pain and it had no effect on her PIP joint swelling. In addition, there is no published report of surgical treatment and it is unknown whether surgical intervention is effective. Therefore, we tried to treat our case with tranilast which inhibits collagen synthesis in human skin fibroblasts [7]. Tranilast is clinically and safely used as an antiallergic drug. Moreover, it is also used as drug for pathological conditions such as keloid formation [15]. We expected the swelling of our patient to go down in light of efficacy of tranilast and histopathological features of pachydermodactyly, which are dermal collagen accumulation and fibroblast proliferation without inflammation. Taking slight improvement of swelling into account, tranilast could be an effective therapeutic agent to pachydermodactyly. However, additional experience is necessary to confirm its possibility.

## 4. Conclusion

We report herein a case of pachydermodactyly treated with tranilast. Although pachydermodactyly is rare and benign, it is important to recognize this condition and be able to differentiate it from other causes of PIP joint swelling such as rheumatoid arthritis. Physicians including orthopaedists and rheumatologists should make a prompt diagnosis to avoid unnecessary investigations and prevent the patient from receiving inappropriate treatment with steroids or cytotoxic agents. On the other hand, tranilast might be an effective drug to pachydermodactyly.
